# A polyaniline/platinum coated fiber optic surface plasmon resonance sensor for picomolar detection of 4-nitrophenol

**DOI:** 10.1038/s41598-021-89396-w

**Published:** 2021-05-12

**Authors:** Iulia Antohe, Iuliana Iordache, Vlad-Andrei Antohe, Gabriel Socol

**Affiliations:** 1 National Institute for Lasers, Plasma and Radiation Physics (INFLPR), Atomiștilor Street 409, 077125 Măgurele, Ilfov Romania; 2 Faculty of Physics, Research and Development Center for Materials and Electronic and Optoelectronic Devices (MDEO), University of Bucharest, Atomiștilor Street 405, 077125 Măgurele, Ilfov Romania; 3 Institute of Condensed Matter and Nanosciences (IMCN), Université catholique de Louvain (UCLouvain), Place Croix du Sud 1, 1348 Louvain-la-Neuve, Belgium

**Keywords:** Surface plasmon resonance, Sensors and biosensors

## Abstract

The paper reports for the first time an innovative polyaniline (PANI)/platinum (Pt)-coated fiber optic-surface plasmon resonance (FO-SPR) sensor used for highly-sensitive 4-nitrophenol (4-NP) pollutant detection. The Pt thin film was coated over an unclad core of an optical fiber (FO) using a DC magnetron sputtering technique, while the 4-NP responsive PANI layer was synthetized using a cost-effective electroless polymerization method. The presence of the electrolessly-grown PANI on the Pt-coated FO was observed by field-emission scanning electron microscopy and subsequently evidenced by energy dispersive X-ray analysis. These FO-SPR sensors with a demonstrated bulk sensitivity of 1515 nm/RIU were then employed for 4-NP sensing, exhibiting an excellent limit-of-detection (LOD) in the low picomolar range (0.34 pM). The proposed sensor’s configuration has many other advantages, such as low-cost production, small size, immunity to electromagnetic interferences, remote sensing capability, and moreover, can be operated as a “stand-alone device”, making it thus well-suited for applications such as “on-site” screening of extremely low-level trace pollutants.

## Introduction

The environmental pollution by phenol-based aromatic nitro compounds in water samples is a major concern worldwide^1^. These nitrophenols are mostly widespread within surrounding environment from industrial wastes, as they are extremely used in the production of pharmaceuticals, pesticides, insecticides, explosives and dyes^2^. According to USA Environmental Protection Agency (EPA), 4-nitrophenol (4-NP) is the most toxic, hazardous and persistent organic pollutant, which can cause significant damages to the health and environment, even at low-level concentrations^3^. Hence, there is need for highly-stable, efficient, robust and reliable sensors that can detect traces of 4-NP, in a rapid and ultrasensitive manner^4^. Until now, several techniques such as capillary electrophoresis, fluorescence, high-performance liquid chromatography (HPLC), mass spectrometry combined with liquid chromatography (LC–MS) or with gas chromatography (GC–MS), and surface enhanced Raman spectroscopy (SERS) have been widely employed for hazardous chemical pollutants sensing, including 4-NP^5,6^. However, these classical analytical techniques have limitations of being time-consuming, and they typically require sophisticated and expensive instrumentation, trained personnel, as well as multistep sample preparation protocols, being thus quite expensive techniques to be commonly used in daily life and industry^7^. In addition, electrochemical approaches such as cyclic voltammetry, linear sweep voltammetry, differential pulse voltammetry and chronoamperometry have similarly shown their potential for the detection of 4-NP^4,8^. Despite the fact that electrochemical methods are generally cost-effective, highly-sensitive and selective, their performance strongly depends on the electrode modifiers, and more important, they are not so stable at temperature and pH fluctuations^9^.

Different from the above-mentioned detection techniques, fiber optic – surface plasmon resonance (FO-SPR) sensing is a relatively novel biochemical method with the advantages of featuring a compact footprint, label-free detection and real-time monitoring capabilities, as well as offering the possibility to perform rapid and non-invasive measurements^10–12^. Such a reflection-type FO-SPR sensor is commonly prepared by uncladding first a small portion at one end of the FO, and then coating the exposed FO core by a plasmonic metal layer, typically gold (Au) or silver (Ag)^13,14^. The state of surface plasmons excited with light (guided by total internal reflection through the FO) at the metal/dielectric surface interface changes when the coated-FO core is immersed within the environment solution containing the target analyte. Thus, a SPR dip at a particular wavelength is then obtained in the reflection spectrum, which strongly depends on the refractive index (RI) of the sensing medium around the metallic layer^10,13^. Owing to that, FO-SPR sensors have been widely used in medical diagnostics and environmental monitoring applications, for studying molecular interactions and their binding specificity^15–17^. For example, Singh et al. reported the development of a transmission-type FO-SPR biosensor for the detection of phenolic compounds (catechol, m-cresol, phenol and 4-chlorophenol) in aqueous solutions^9^. The sensing probe was prepared by depositing Ag film onto FO core via a thermal evaporation method followed by the immobilization of enzyme tyrosinase, using a gel entrapment technique. In this case, the authors claimed a limit-of-detection (LOD) for all analyzed phenolic compounds in the low µM concentrations range. Alternatively, Cennamo et al. presented a detection scheme of another nitrophenol compound (TNT-2,4,6-trinitrotoluene) based on the combined approach of FO-SPR and molecular imprinting technique^18^. The SPR device was obtained by coating a 60 nm thick Au film over the FO core using a sputtering method. The sensing method demonstrated a detection limit of 51 μM with a sensitivity of 27 μm/M. However, one year later the authors have shown further improvements of the TNT sensor by designing a localized SPR (LSPR) device, through the incorporation of branched Au nanostars dispersed into a molecular imprinted polymer initially coated on the FO core. In this way, the authors obtained better LOD and sensitivity values, of 2.4 μM and 84 μm/M, respectively^19^. Noteworthy, to the best of our knowledge, yet there is no evidence in literature of employing a reflection-type FO-SPR sensor for 4-NP detection.

In this work, results on the fabrication and characterization of an innovative FO-SPR sensor, based on a polyaniline (PANI) / platinum (Pt) bilayer coated over an unclad FO core, and used for 4-NP detection, were reported for the first time. The Pt thin film was deposited by DC magnetron sputtering and it replaced the conventional Au layer commonly preferred with a traditional reflection-type FO-SPR sensor^13^. So far, only a limited number of theoretical studies were reported with Pt-coated FO-SPR sensors operating in transmission mode^20,21^. Herein, using Pt as a plasmonic material within reflection-type FO-SPR sensors is a novel approach and the excellent catalytic properties of Pt are essential for subsequent PANI synthesis steps^22,23^. Complementarily, PANI is an organic polymer with excellent stability and physico-chemical properties in terms of high electrical conductivity, large electro-active surface, and unique combination of RedOx states and proton doping profiles^4,24^. These particular features render PANI as an extremely responsive polymer to several molecular species, being so far successfully used in energy storage applications^25^, pH monitoring^26^, gas sensing^27^ and pollutants detection including nitrophenol compounds^4^. In this work, PANI was synthetized using a cost-effective electroless polymerization approach, in an attempt to uniformly deposit thin sensitive PANI films on the curved Pt-coated FO core three-dimensional (3D) geometry. The PANI/Pt-based FO-SPR sensor was then morphologically characterized and evaluated for highly-sensitive 4-NP pesticide detection in water samples, demonstrating a sensor’s LOD in the low pM concentrations range. This work represents thus a step forward in the fabrication of reliable FO-SPR sensors, not only with improved performance, but also with extended functionality.

## Materials and methods

### Reagents and materials

All the reagents used in this work were of analytical grade (99.99% purity, unless otherwise specified). Ultra-clean deionized water (DIW), purified by a TKA Milli-Q 50 system, was consistently used throughout the experiments. Acetone, sulfuric acid (97% H_2_SO_4_), D( +)-sucrose, ethanol and aniline (99% C_6_H_5_NH_2_) were supplied by Merk. The nitrogen and oxygen 5.0 purity gas bottles were acquired from Messer. The TEQS multimode FO of 400 μm diameter was provided by Thorlabs. The additional tools used in the aniline polymerization protocol, such as the Pasteur glass pipettes (2 mL capacity, 230 mm length), the double-wall glass (diameter 55 mm, capacity 150 mL, height 85 mm) and the magnetic stir plate were obtained from VWR. The TC120 heated circulating bath was purchased from Grant Instruments.

### FO-SPR setup and sensors fabrication

**Figure 1:**
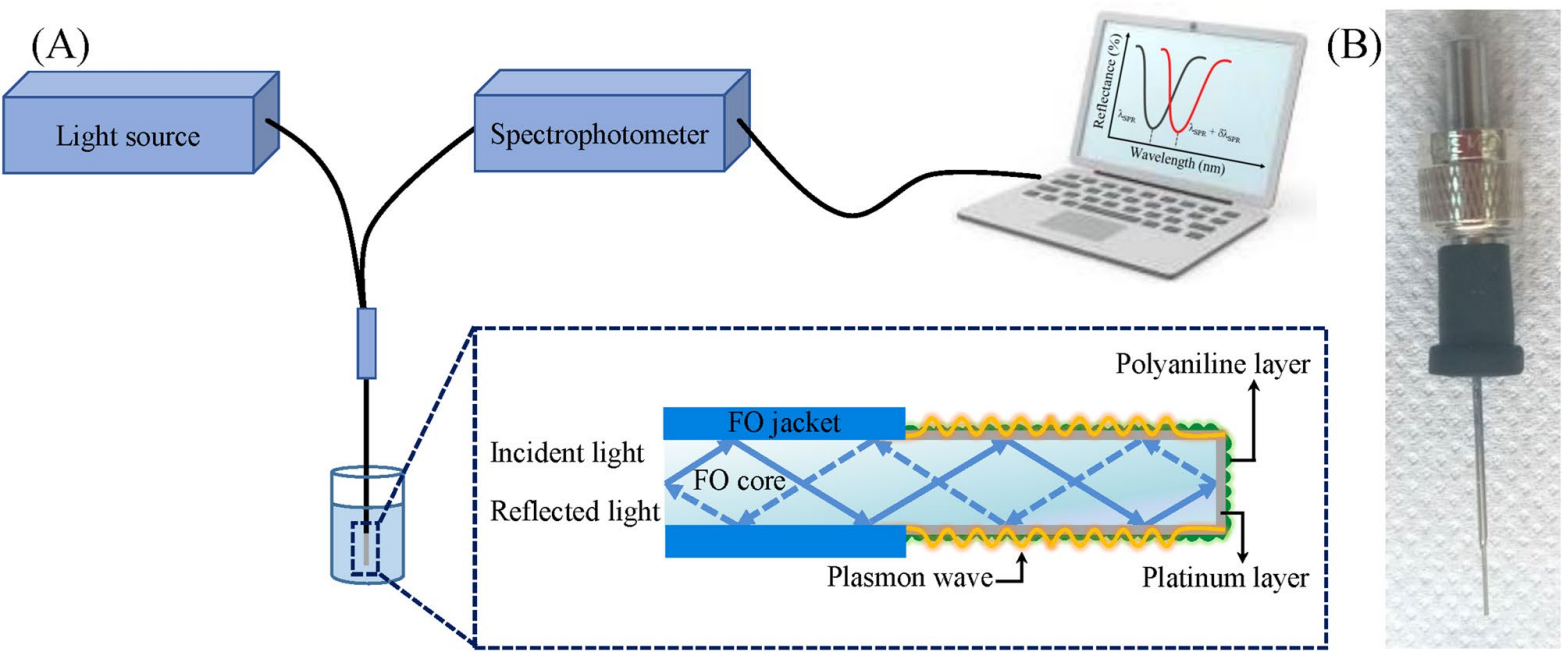
FO-SPR sensing platform. (**A**) Schematic of the experimental setup; (**B**) Image of the fabricated Pt-coated FO-SPR sensor inserted into the SMA connector. Designed using PowerPoint 2016 (Microsoft Office 2016), https://www.microsoft.com/.

The “in-house” developed FO-SPR sensing platform consists of several components, as illustrated in Fig. 1A: a polychromatic tungsten halogen light sources (AvaLight, Avantes), an UV–VIS spectrophotometer (AvaSpec 2048, Avantes), an interchangeable FO-SPR sensor (Fig. 1B) inserted into a SMA (SubMiniature version A) connector (Avantes) and mounted in a bifurcated FO (Avantes), as well as an automated computer-controlled robotic arm programmed using the ColiDrive software (Colinbus). The light passing through the SPR sensitive zone is reflected back at the FO sensing tip and measured using the spectrometer. Any change in the surrounding environment occurring at the Pt surface results in a shift of the typical SPR spectral resonance dip, subsequently monitored in real-time and processed using an “in-house” developed LabVIEW script (National Instruments). The interchangeable FO-SPR sensors were prepared using a previously described protocol^13,28^. In brief, the multimode FO with a diameter of 400 μm was first split into 3.6 cm long segments. Then, a sensitive SPR zone of 0.6 cm was constructed at one side by mechanically removing the jacket and subsequently uncladding the FO in acetone. The exposed FO silica core was then carefully dried with dust-free tissues and under N_2_ gas flow. Next, the sensor tips were isotropically coated by a thin Pt layer (40 nm) using a sputter coater (Quorum Q150R ES, UK). The DC plasma was engaged for 15 min at 54 mA in an Ar atmosphere kept at 2.5 Pa. The FO-SPR sensor tips were installed on a rotating stage (100 rpm) to improve the Pt FO coverage during the sputtering process, while the deposited thickness was monitored using the built-in quartz crystal oscillator (QCM). The reliability of the Pt layer thickness covering the FO-SPR sensor tips and its evenness were thus assured by the well-known isotropic nature of the sputtering process, coupled with the accuracy of the QCM real-time measurement. Ultimately, the Pt coated-FO sensor tips were used as catalysts in the electroless polymerization process of aniline.

### FO-SPR refractometric measurements

The bulk sensitivity of the Pt-coated FO-SPR sensors was evaluated by performing refractive index (RI) measurements in sucrose dilutions (0, 2, 4, 8, 12% w/w). The Brix values of the prepared sucrose dilutions were checked with a digital refractometer (Atago Palette PR-32) and their corresponding RI values are presented in Table 1.

**Table 1:** RI values of different sucrose dilutions (0% stands for DIW).

Sucrose (%)	0	2	4	8	12
RI	1.3330	1.3359	1.3388	1.3447	1.3509

The interchangeable FO-SPR sensors were inserted into the SMA connectors and then mounted on the computer-controlled SPR measuring platform. The Pt-coated FO-SPR sensors were kept one minute in each sucrose solution during the RI measurements. Each sensor was used to measure once the serial set of sucrose dilutions (0, 2, 4, 8, 12% w/w). The FO-SPR bulk sensitivity was afterwards evaluated by plotting the SPR wavelength shifts as a function of the RI values of each sucrose solution, followed by linearly fitting the obtained calibration curve. Furthermore, using the data analysis and graphing Origin software package (OriginLab), the figure of merit (FOM) for the fabricated FO-SPR devices was also determined by making the ratio between the wavelength shift bulk sensitivity (S) and the linewidth of the spectral resonance dip, given as the full width at half-maximum (FWHM), being expressed in [RIU^−1^], where RIU stands for Refractive Index Unit. The FOM is thus an important quality parameter, as it quantifies the degree of FO-SPR sensor’s effectiveness.

### The PANI electroless deposition method

The basic steps of PANI electroless deposition methodology were taken from literature^25,26,29,30^ and carefully adapted to the micrometer-sized curved FO 3D geometry. Generally, the clean Pt covered substrates were immersed in an aqueous solution of aniline (0.4 M) and H_2_SO_4_ (0.4 M) for 2, 4 and 6 h, respectively. The solution was maintained under constant oxygen gas flow (kept at 0.5 sccm) and at a constant temperature of 25 °C for reproducibility purposes, as it was previously reported that PANI electroless growth rate is extremely sensitive to temperature fluctuations^29^. A green film gradually grew on the Pt surface, being an indication for the formation of a PANI mid-RedOx state close to the Emeraldine (EM) salt, as previously mentioned^25^. After a given immersion time, the sensors were taken out of the electroless reactor and thoroughly washed with DIW. Before employing the as-prepared sensors for 4-NP sensing, the obtained PANI films were undoped with a 1 M NH_4_OH solution for 10 min to induce an initial well-known Emeraldine base state of PANI, marked by a change in the film color from light green to dark blue.

### Observations of the FO-SPR surfaces and PANI thickness measurement

A field-emission scanning electron microscope (FE-SEM, JEOL7600F) equipped with an energy dispersive X-ray (EDX) analyzer was used to investigate the surface morphology and structural properties of the PANI film deposited on the Pt-coated FO-SPR sensors. A low accelerating voltage (2 kV) was constantly applied to reduce the charging effects and to extract more information close to the sample surface. The EDX spectroscopy was effectively used to qualitatively and quantitatively confirm the elemental composition of the fabricated FO-SPR sensors. Noteworthy, SEM and EDX analysis were carried out on Pt-coated FO-SPR sensors covered by PANI films intentionally doped in a 1 M HCl solution for 10 min to enhance their conductivity and hence to further reduce specimens charging during the SEM observations.

In a second stage, the PANI film thickness was determined by profilometry (Stylus Profiler XP-2, Ambios Technology), providing precision surface topography measurements with 1.5 Å vertical resolution. In this case, the Pt-coated FO-SPR sensor tips were half-covered with an adhesive tape before their immersion into the electroless reactor, in order to generate a Pt-PANI height-step profile after the tape subsequent removal. The FO-SPR sensors were horizontally positioned on the profilometer specimen holder and the profilometer tip was moved on top of the cylindrical FO side about 400 μm across the Pt-PANI height-step profile.

### Detection of 4-nitrophenol in water samples

The as-prepared PANI/Pt-coated FO-SPR sensors were further used for direct and subsequent detection of different concentrations of 4-NP (0, 1, 100, 10^3^, 10^5^ and 10^6^ pM) in DIW. Each 4-NP concentration was measured three times independently, using freshly prepared PANI/Pt-coated FO-SPR sensors.

## Results and discussion

### Bulk sensitivity of the Pt-coated FO-SPR sensors

The bulk sensitivity performance of the Pt-coated FO-SPR sensors was evaluated by performing RI measurements in serial sucrose dilutions. As aforementioned already, despite few theoretical attempts^20,21^, this work reports for the first time on the fabrication of a reflection-type FO-SPR sensor based on a Pt plasmonic layer and the determination of its performance indicators (i.e., bulk sensitivity—S and figure of merit—FOM).

**Figure 2:**
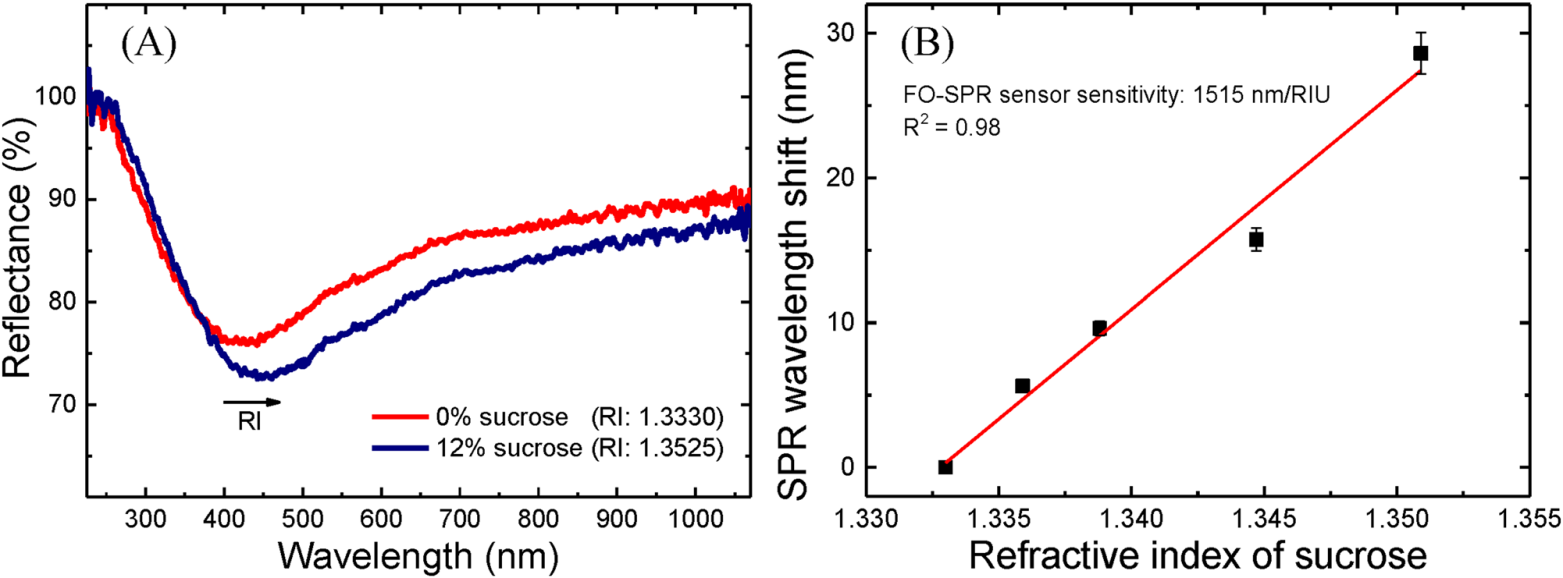
Evaluation of the Pt-coated FO-SPR sensors performance. (**A**) SPR spectral dips obtained at 0 (red) and 12% (blue) sucrose concentrations; (**B**) Corresponding calibration curve measured at sucrose concentrations (0, 2, 4, 8 and 12% w/w) with the Pt-coated FO-SPR sensors. The error bars represent standard deviation (n = 5) and R^2^ denotes the coefficient of determination. Processed using Origin 9.6.0.172 (OriginLab 2019), https://www.originlab.com/.

Figure 2A shows the SPR spectral dips obtained at 0 (red curve) and 12% (blue curve) sucrose concentrations. The obtained SPR shifts were plotted as a function of RI values for generating the calibration curve presented in Fig. 2B. The bulk sensitivity values were then extracted from the slope of these calibration curves. In this way, the bulk sensitivity was determined to be around 1515 nm/RIU. In the case of FO-SPR sensors, the bulk sensitivity (S) is expressed as the ratio between the wavelength shift (Δλ_SPR_) and the RI change (Δn) in the analyzing medium: S = Δλ_SPR_/Δn [nm/RIU]^10^. Furthermore, the FOM of the Pt-coated FO-SPR sensors was also calculated to be around 7 RIU^−1^. Table 2 gives a brief performance comparison among various types of FO-SPR sensors reported in literature.

**Table 2:** Performance comparison data between different FO-SPR sensors.

FO-SPR sensor’s configuration	RI measurements	Bulk sensitivity (nm/RIU)	FOM (RIU^−1^)
Au-coated FO-SPR with Ag mirror coated on the FO tip^41^	Ethanol dilutions	1557	ca. 19.4
FO-LSPR sensor with the FO tip coated by both, Au and Ag nanoparticles^42^	Glycerol dilutions	390	ca. 6.5
Au-coated FO-SPR sensor^31^	Sucrose dilutions	1520	ca. 19
Pt-coated FO-SPR sensor [this work]	Sucrose dilutions	1515	ca. 7

As can be noticed, the obtained Pt-coated FO-SPR sensor’s bulk sensitivity was similar with the one of the Au-coated FO-SPR sensor previously reported^31^, while the FOM was lower, mainly due to the higher FWHM value. Although the specificity and selectivity of Pt-based FO-SPR sensors may suffer due to the more broad SPR spectral dips observed, under optimized preparation protocols their bulk sensitivity value can compete with the traditional Au-coated reflection-type FO-SPR sensors. Besides, the Pt-based FO-SPR sensors may extend the applicative range of such optical sensing devices, by benefiting from the Pt chemical catalytic activity and stability, or by shifting the operational spectral range towards lower wavelengths.

### PANI deposition on the Pt-coated FO-SPR sensors

PANI thin films were synthesized on the Pt-coated FO-SPR substrates using a relatively novel electroless deposition method well described in literature^29,30^, where PANI is simply obtained through the polymerization of aniline on the Pt surface acting as a catalyst. The process is based on spontaneous chemical reactions in acidic medium, involving reduction of dissolved oxygen as cathodic half-reaction and oxidation of aniline as anodic half-reaction at the metal/solution interface^29^. The polymerization reaction is thus initiated on the Pt surface by a catalytic oxygen reduction, and then the primary formed PANI layer takes over the autocatalytic polymerization of aniline. Consequently, when the Pt-coated FO-SPR sensors were immersed in the electroless reactor kept under oxygen saturation, a light greenish color gradually appeared on their surface. The greenish color appearance is a characteristic of the acidified Emeraldine mid RedOx state of PANI. In this work, the thickness of the electrolessly-grown PANI film on the Pt-coated FO-SPR sensor was also studied as a function of the reaction time. The PANI thickness was accurately evaluated by profilometry.

**Figure 3:**
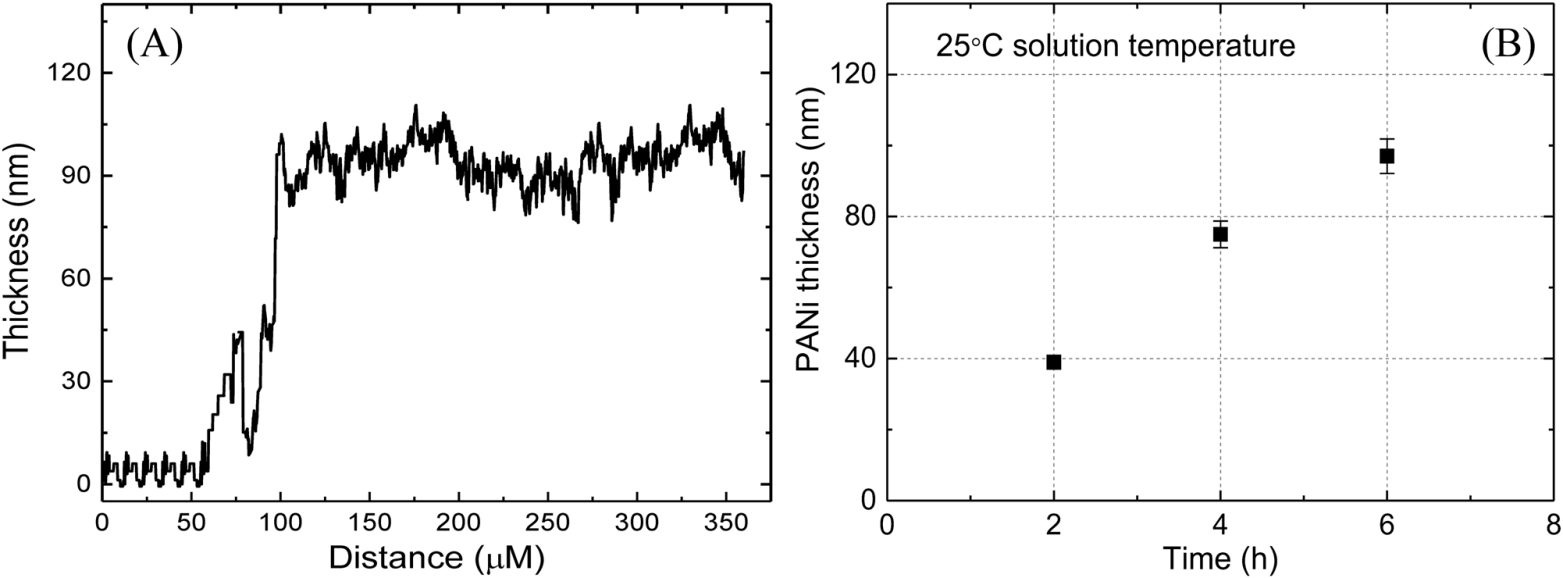
PANI thickness obtained for different reaction times. (**A**) Typical profilometric measurement taken on a PANI/Pt-coated FO-SPR sensor kept for 6 h in the reactive medium, showing a PANI thickness of ~ 95 nm. (**B**) Thickness of electrolessly-deposited PANI films obtained in the equimolar (0.4 M) solution of aniline and H_2_SO_4_ at 25 °C, after 2, 4 and 6 h, respectively. The error bars represent standard deviation (n = 3). Processed using Origin 9.6.0.172 (OriginLab 2019), https://www.originlab.com/.

Several Pt-coated FO-SPR sensors were immersed in the equimolar (0.4 M) aqueous solution of aniline and H_2_SO_4_ kept at 25 °C under continuous oxygen bubbling, and gradually removed after 2, 4 and 6 h, respectively. The thickness of the grown PANI film after each immersion duration was measured using surface profilometry. As can be observed in Fig. 3B a linear time-dependence of the PANI thickness was found, as previously reported^30^. This signifies that the polymerization rate is constant, PANI growth occurring at a rate of ~ 17 nm/h. A typical example of profilometric measurement after 6 h PANI growth is shown in Fig. 3A, where the height-step of ~ 95 nm between the Pt-coated FO-SPR surface and the PANI film denotes the thickness of the latter.

### Morphological and structural characterization of the FO-SPR surface

**Figure 4:**
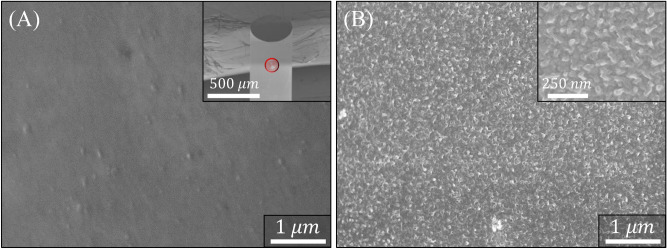
FE-SEM micrographs of the Pt-coated FO-SPR surface before (**A**) and after (**B**) PANI electroless deposition for 6 h at 25 °C. The inset of (**A**) low-magnification SEM image of the FO-SPR sensor tip where the red mark denotes the area where the higher-magnification SEM images (**A**,**B**) were captured; The inset of (**B**) corresponding closed-up magnification SEM image of the PANI/Pt-coated FO-SPR sensor surface. For SEM observations, the conductivity of the PANI film was slightly enhanced by protonating it for 10 min in a 1 M HCl solution. Processed using PowerPoint 2016 (Microsoft Office 2016), https://www.microsoft.com/.

FE-SEM micrographs acquired from the surface of a Pt-coated FO-SPR sensor before and after PANI electroless deposition for 6 h at 25 °C are shown in Fig. 4. As can be observed, the Pt-coated FO-SPR sensor has a homogenous and smooth surface (Fig. 4A). Noteworthy, the FO-SPR sensors (inset of Fig. 4A) were investigated in several places, with similar results obtained for both the FO tip and its circular sides, as expected due to the FO-SPR sensors rotation during the deposition step and isotropic nature of the DC magnetron sputtering process^13,28^. Similarly, the PANI film on the Pt-coated FO-SPR sensor (Fig. 4B) is evidenced by the roughened curly aspect of the surface (magnified in the inset of Fig. 4B), typical for a thin PANI film grown on a Pt substrate through an electroless synthesis procedure^29,32^. As can be noticed, the obtained PANI film was homogeneous, dense, with good conformality and well adhered to the Pt-coated FO-SPR surface. This wavy aspect of the PANI surface was found to be more pronounced for the 6 h electroless synthesis duration and it is believed that it plays an important role in the FO-SPR sensing performance^28^, contributing to the reason of selecting the ~ 95 nm PANI thickness as optimal for subsequent 4-NP detection studies.

**Figure 5:**
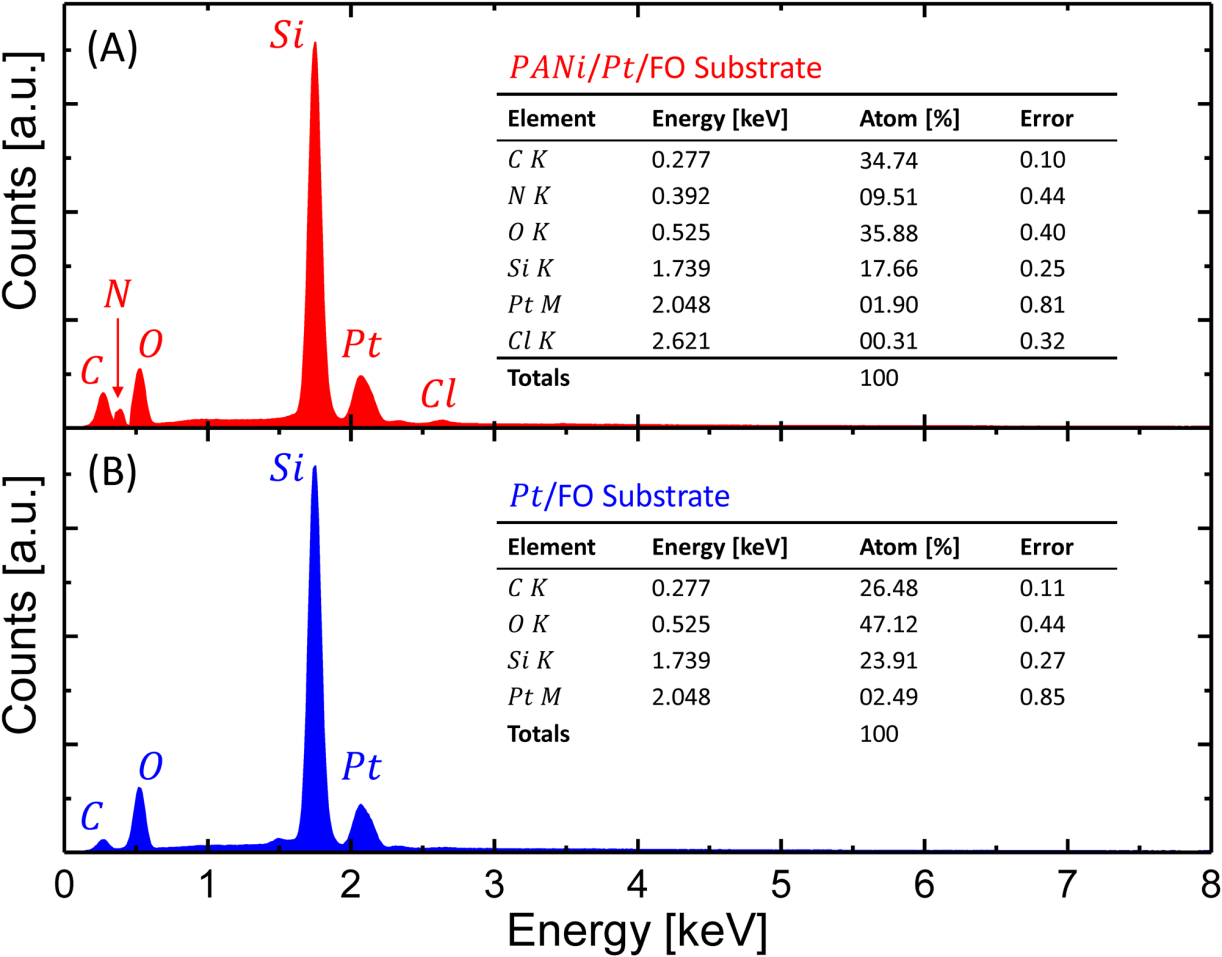
EDX analysis of the Pt-coated FO-SPR sensor surface, after (**A**) and before (**B**) PANI electroless deposition for 6 h at 25 °C. The insets show corresponding quantitative compositional calculation charts. Processed using Origin 9.6.0.172 (OriginLab 2019), https://www.originlab.com/.

Corresponding EDX patterns of the FO-SPR surface presented in Fig. 4 are shown in Fig. 5. The EDX analysis performed on the Pt-coated FO-SPR surface (Fig. 5B) well indicates the presence of the constitutive elements, i.e. oxygen (O), silica (Si) and Pt. The small intensity of the carbon (C) peak in the Pt-coated FO-SPR surface can be attributed to the adhesive carbonic conductive tape used to fix the sensors during the FE-SEM/EDX measurements. In addition, the PANI presence on the Pt-coated FO-SPR surface is suggested by a nitrogen (N) peak and a higher intensity carbon (C) peak, respectively (Fig. 5A). However, small chlorine (Cl) traces could be also observed in Fig. 5A due to the PANI protonation step in HCl. The insets of Fig. 5A,B show associated quantitative elemental calculation charts, where the results described well the coexistence of both, supporting Pt-coated FO silica core (i.e. Pt/SiO_2_) and PANI film.

### Detection of 4-nitrophenol in water samples

**Figure 6:**
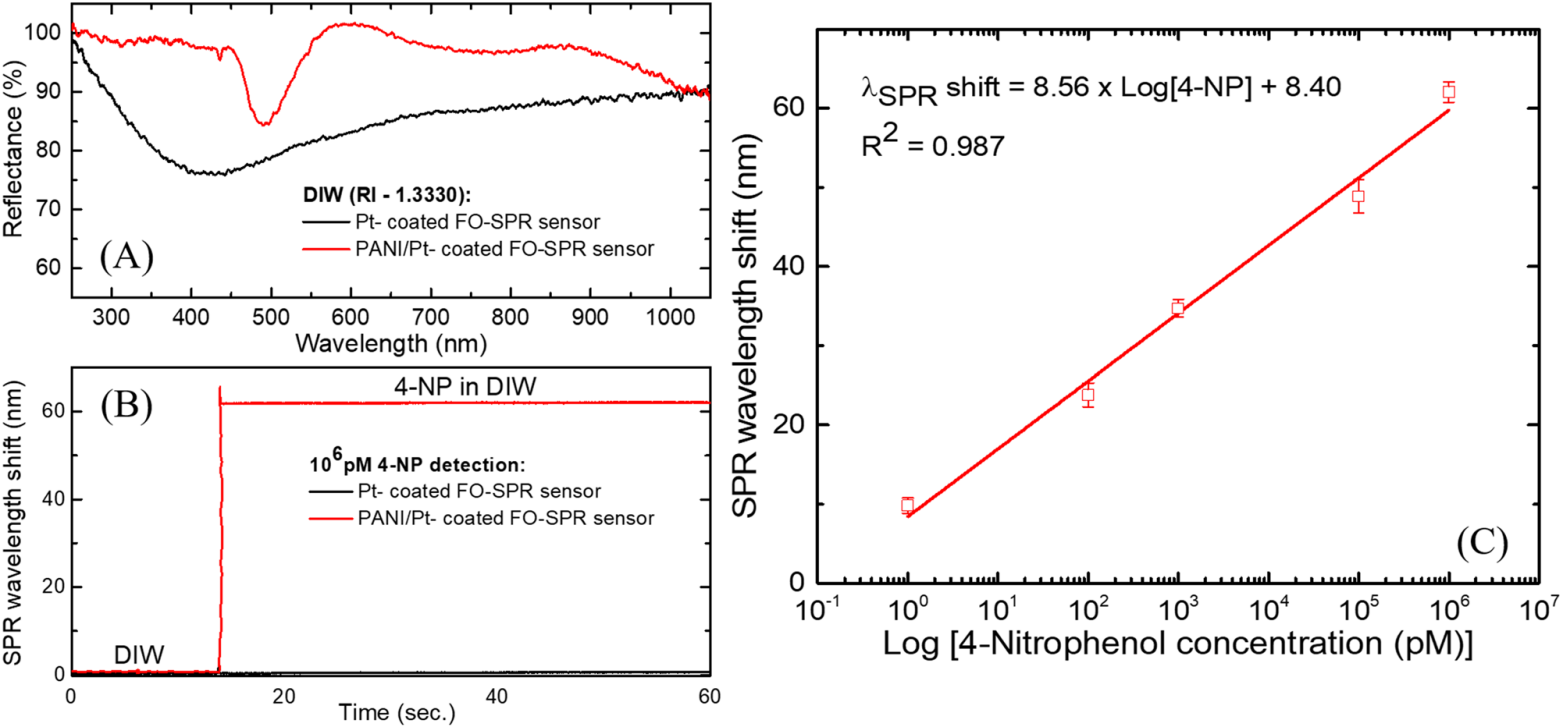
Detection of 4-NP in DIW samples. (**A**) SPR spectral dips obtained in DIW with Pt-coated (black) and PANI/Pt-coated (red) FO-SPR sensors, respectively; (**B**) Specificity test, showing that in the absence of PANI layer a SPR wavelength shift of less than 1 nm is recorded for the solution containing 10^6^ pM 4-NP in DIW; (**C**) Calibration plot showing a linear relationship between the PANI/Pt-coated FO-SPR wavelength shift and the logarithm of 4-NP concentrations. The error bars indicate the standard deviation (n = 3). Processed using Origin 9.6.0.172 (OriginLab 2019), https://www.originlab.com/.

At this stage, PANI/Pt-coated FO-SPR sensors (prepared as described in the previous sections) have been tested in DIW to evaluate their SPR spectral response, when compared to the bare Pt-coated ones. A value of the PANI thickness around 95 nm was found to be optimal, as highlighted in Fig. 6A showing the corresponding SPR spectral dips obtained in DIW with the Pt-coated (black) and PANI/Pt-coated (red) FO-SPR sensors, respectively. As can be noticed, a narrower SPR spectral dip shifted towards longer wavelengths was observed after PANI electroless polymerization on the surface of the Pt-coated FO-SPR sensor, due to the expected changes of dielectric properties of the PANI film when exposed to the analyzing media^33,34^. The as-prepared PANI/Pt-coated FO-SPR sensors (with ~ 95 nm PANI thickness) were then used to detect five concentrations of 4-NP (0–10^6^ pM range) in DIW samples. The FO-SPR sensor specificity was first tested by employing a Pt-coated sensor (without PANI layer) for detecting the highest 4-NP concentration in DIW (10^6^ pM). As shown in Fig. 6B, although insignificant, a SPR wavelength shift of less than 1 nm can be observed, possibly due to a very slight change in the RI of the 4-NP-reach analyzing medium, and/or due to absorption effects of the 4-NP molecules on the Pt surface in the absence of the sensitive PANI film.

**Figure 7:**
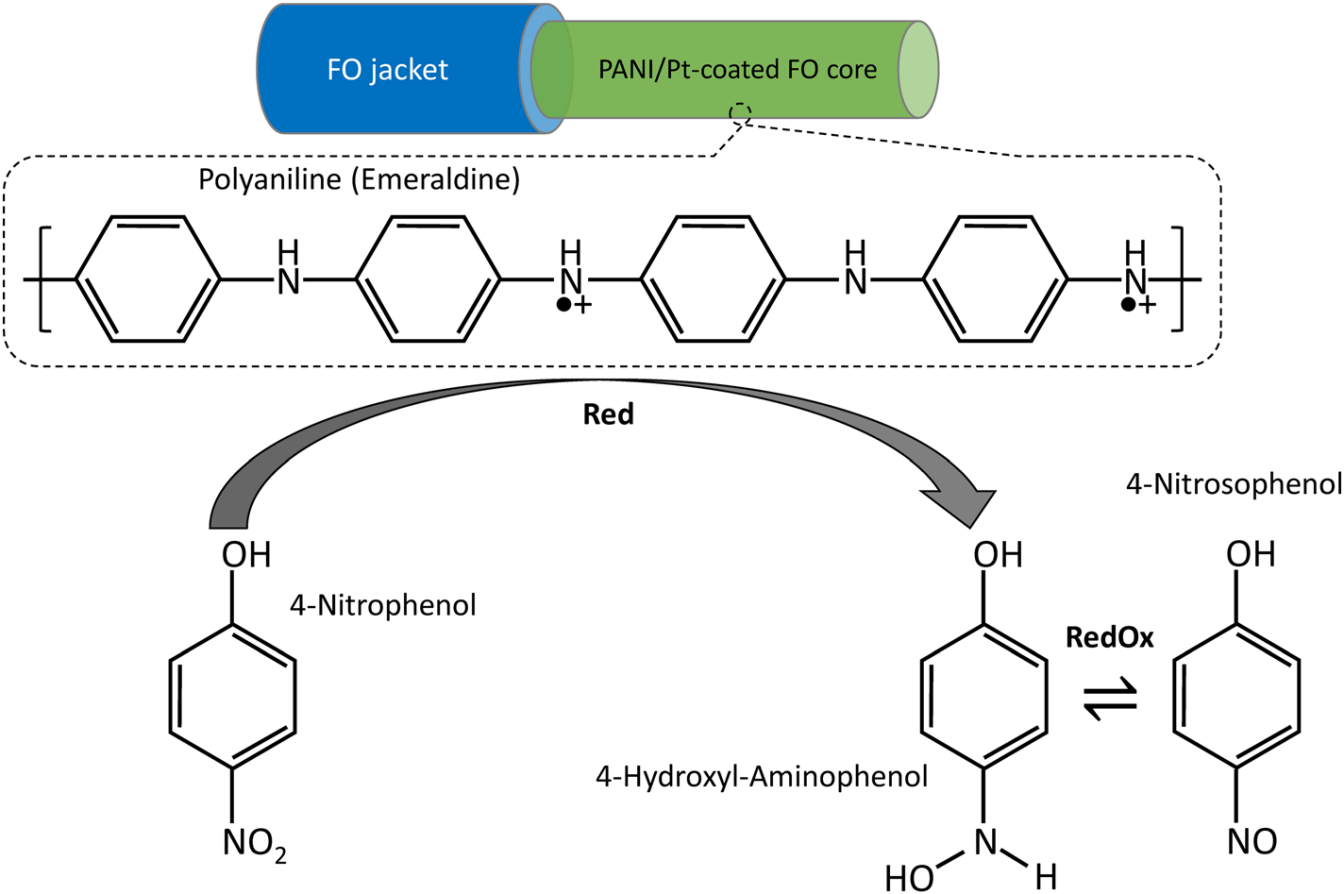
Proposed sensing mechanism of the 4-NP with the PANI/Pt-coated FO-SPR sensor, based on the mediated PANI/Pt catalytic activity of 4-NP reduction. Designed using PowerPoint 2016 (Microsoft Office 2016), https://www.microsoft.com/.

Figure 6C shows the linear-log plot acquired after 1 min detection of each 4-NP concentration with the PANI/Pt-coated FO-SPR sensor. The linear relationship was given by a regression equation with a coefficient of determination (R^2^) of 0.987 and a slope (sensor’s sensitivity defined as the shift of the SPR wavelength per unit change in the logarithm concentration of 4-NP) of 8.56 nm/Log (pM)^16,35^. Furthermore, the LOD was estimated according to the “3σ rule” (3 × σ/S, where σ represents the standard deviation of the lowest concentration measured and S is the slope of the calibration curve), leading to a promising LOD value of 0.34 pM (or equivalently, 4.72 × 10^–11^ µg/mL)^10,16,28,35–37^. The sensing mechanism can be generally explained in terms of a PANI-mediated process in which H^+^–terminated sites of PANI trigger the reduction of 4-NP to 4-hydroxyl-aminophenol, followed by subsequent oxidation of the latter to yield 4-nitrosophenol (see Fig. 7)^7,38^. Consequently, the catalytic properties of PANI/Pt bilayer take over a RedOx reaction, generating important changes within medium’s RI through 4-NP conversion into 4-nitrosophenol, and causing further sensitive shifts within SPR spectral dips wavelength position, as observed within the calibration curve presented in Fig. 6C. These excellent performance indicators are also a consequence of an optimal PANI thickness (i.e. ~ 95 nm) and of its particular roughened curly-like superficial morphology (Fig. 4B) that generates an overall increase of the FO-SPR sensor active surface^28^, thus potentially providing a more efficient catalytic surface reaction between 4-NP and PANI film.

Table 3 shows a synopsis of few common methods employed so-far for 4-NP detection. As can be noticed, Guo et al. were able to determine 4-NP with rather good results (a LOD of 4.06 μM, although for a limited high μM linear range)^6^. However, Tang et al. demonstrated better results when employing an electrochemical sensor operating in differential pulse voltammetry (DPV) mode (a LOD of 10 nM, with a low μM linear range)^39^, followed by the studies of Manera et al. demonstrating a LOD of only 3 nM for a similar linear range when employing a diffuse reflectance optosensing technique with multivariate regression modelling for trace-level determination of 4-NP^40^. Nevertheless, it can be noticed that in this work, the calculated LOD value of 0.34 pM is three orders of magnitude lower compared to the letter approach for a concentrations range well-bellow 1 μM. Consequently, the PANI/Pt-coated FO-SPR sensors developed and characterized in this work could be potentially used for highly-sensitive detection of very low trace-level 4-NP pesticides within various media.

**Table 3:** Synopsis of the 4-NP detecting performance by alternative sensing methods.

Sensing method for 4-NP detection	Concentrations range (μM)	LOD (μM)
High performance capillary zone electrophoresis (HPCZE)^6^	20.3–4060	4.06
Electrochemical sensor, differential pulse voltammetry (DPV) detection mode^39^	0.05–2	0.01
Diffuse reflectance optosensing^40^	0.007–0.6	0.003
PANI/Pt-coated FO-SPR sensor [this work]	10^–6^–1	3.4 × 10^–7^

## Conclusions

In this work, an innovative PANI/Pt-coated FO-SPR sensor was used to determine the amount of 4-NP in DIW samples. The sensing area was fabricated by coating the uncladded FO core with Pt and subsequently depositing a thin sensitive PANI film. The Pt plasmonic layer was evenly coated on the cylindrical FO silica core by DC magnetron sputtering and afterwards used as catalyst for the uniform and conformal polymerization process of PANI via a simple cost-effective electroless procedure. The bulk sensitivity of the as-prepared Pt-coated FO-SPR sensors was first evaluated in serial sucrose dilutions (of different RI units), owning to a value of 1515 nm/RIU, comparable with the traditionally-reported Au-coated FO-SPR sensors. Second, the PANI/Pt-coated FO-SPR sensors unveiled encouraging results when employed for 4-NP detection, as the LOD was estimated to 0.34 pM, with a sensitivity of 8.56 nm/Log (pM), excellent performances obtained so far in respect with previous literature reports. These PANI/Pt-coated FO-SPR sensors may provide a broad interest for applications, especially in highly-sensitive real-time detection of extremely low trace-level pollutants.
